# Facing Dementia in Primary Care: Applying the COM-B Model to Develop a Complex Intervention to Improve Dementia Diagnosis Rates in General Practice

**DOI:** 10.3390/ijerph23050653

**Published:** 2026-05-14

**Authors:** Caroline Gibson, Mark Yates, Constance Dimity Pond, Stephanie Daly, Jessica Jebramek, Lyn Phillipson, Kate Laver, Meredith Gresham, Edwin Tan, Henry Brodaty, Jamie Swann, Shahana Ferdousi, Lee-Fay Low

**Affiliations:** 1 School of Medicine, Faculty of Health, Deakin University, Ballarat, VIC 3350, Australia; 2 Wicking Dementia Research and Teaching Centre, University of Tasmania, Hobart, TAS 7001, Australia; 3 Australian Centre for Evidence Based Aged Care, Latrobe University, Melbourne, VIC 3000, Australia; 4 Faculty of the Arts, Social Sciences and Humanities, University of Wollongong, Wollongong, NSW 2500, Australia; 5 College of Nursing and Health Sciences, Flinders University, Adelaide, SA 5000, Australia; 6 School of Pharmacy, Faculty of Medicine and Health, University of Sydney, Sydney, NSW 2000, Australia; 7 Centre for Healthy Brain and Aging, University of New South Wales, Sydney, NSW 2000, Australia; 8 School of Health and Social Development, Health Services and Digital Innovation, Deakin University, Burwood, VIC 3125, Australia; 9 Centre for Health Research, Western Sydney University, Sydney, NSW 2000, Australia; 10 School of Health Sciences, Faculty of Medicine and Health, University of Sydney, Sydney, NSW 2000, Australia; lee-fay.low@sydney.edu.au

**Keywords:** dementia diagnosis, primary care, general practitioner, behaviour change theory, complex intervention, practice change, co-design

## Abstract

**Highlights:**

**Public health relevance—How does this work relate to a public health issue?**

- Dementia represents a significant public health challenge, as it is one of the major causes of death, disability and dependency among older people worldwide.
- Dementia is often not diagnosed, and dementia action plans have identified the need to support general practitioners in dementia diagnosis and care.

**Public health significance—Why is this work of significance to public health?**

- Historically, specialists have been responsible for dementia diagnosis; this work explores how to share some of this responsibility with primary care.

**Public health implications—What are the key implications or messages for practitioners, policy makers and/or researchers in public health?**

- A public health approach to increasing timely diagnosis of dementia requires a co-designed, theory-informed, multi-component primary care practice change program.
- The intervention needs to extend beyond education and training to address barriers to dementia diagnosis and promote behaviour change.

**Abstract:**

As the population ages and new therapies become available, general practitioners will have a significant role in the early detection, diagnosis, and management of dementia. However, both in Australia and globally, dementia remains under-recognised and under-diagnosed in primary care. The aim of this study is to develop a complex intervention, informed by behaviour change theory, to improve rates of dementia diagnoses in Australian primary care. Co-design participants included GPs, general practice nurses, practice managers and reception staff. A program logic model was used to describe the essential activities and mechanisms of the intervention. Six behaviour changes—education, training, enablement, modelling, persuasion, and environmental restructuring—were identified to address the identified barriers to dementia diagnosis in primary care. The intervention comprises seven activities—peer-led online dementia education and training, geriatrician ‘drop-in’ online support sessions, quality improvement in dementia care sessions, stand-alone videos, auditing and benchmarking, a dementia risk alert tool and a set of dementia diagnosis and management decision-making resources. Using behaviour change theory can assist in the development of complex interventions aimed at changing clinical practice and may assist in their evaluation.

## 1. Introduction

Optimal care and management of individuals living with dementia and their carers have been shown to mitigate the impact of symptoms and help maintain independence within the community for as long as possible [1]. The core principles of primary care include enhancing population health through health promotion and disease prevention, fostering long-term patient relationships, and providing coordinated, person-centred care [2]. These principles position primary care to deliver cost-effective healthcare and potentially more holistic support for people living with dementia compared to secondary care settings [3]. Consequently, as the population ages and new therapies become available, primary care is expected to assume a significant role in the early detection, diagnosis, and ongoing management of dementia [4].

Although general practitioners (GPs) are well-positioned to diagnose dementia and manage patient care [2], dementia remains under-recognised and under-diagnosed both in Australia and globally [5]. Documentation of a dementia diagnosis in primary care is lower than expected—68% of that reported in the Australian Census data and 48% of population estimates [6]. People living with dementia and carers report low satisfaction with GP care [7], and that GPs are reluctant to diagnose dementia [8] and often dismiss concerns [9].

Practitioner, community and health system level barriers to the diagnosis and management of dementia in primary care are well documented [7,10,11,12,13]. Individual barriers from the perspective of GPs include diagnostic uncertainty [14], the belief that the negative consequences of diagnosis (impact on self-image and experiences of stigma) outweigh the modest effects of symptomatic treatments [15,16], and a perceived lack of support options for patients following a diagnosis [1,7]. Barriers from the patient and family perspective include a lack of awareness by older people and families of dementia symptoms, or denial, and delayed help-seeking [9]. Institutional barriers include a lack of time and funding [10,17] and limited communication between GPs and specialists [18]. Strong interprofessional relationships and team-based care have been found to support GPs’ willingness to initiate conversations about dementia and start the diagnostic process [15].

Within Australia, and internationally, dementia action plans have identified the need to support GPs in their delivery of dementia care and have advocated the development and roll-out of dementia educational programs for GPs [19,20,21,22]. However, research on educational interventions for dementia shows mixed effects on GPs’ knowledge and confidence, with minimal demonstrated impact on clinical practice [23,24,25]. Guidelines and education increase knowledge but may not change motivations or opportunities. Similarly, introducing new policies such as financial incentives shows mixed results in bringing about practice change [26]. Instead, multi-component approaches are often necessary to effect a change in clinical practice [27] Globally, a variety of primary care models that combine education with additional strategies have been implemented to offer support to the GP to provide better dementia care for people. These models of care include case management models [28,29,30], integrated geriatrician-led memory clinics [31] and access to specialist dementia care nurses [32]. However, these models have had varying outcomes and have not been theoretically informed.

The Medical Research Council framework [33] emphasises the importance of a theoretical approach in the development and evaluation of complex interventions. Complex interventions refer to health or social care strategies that include multiple interacting elements and are designed to target multiple behaviours or operate across different levels, such as individuals or organisations [34]. Clinical practice, as a form of human behaviour, can be explained using behaviour change theories, which, in turn, can provide the foundation for systematically designing complex interventions aimed at clinical practice change [35].

The COM-B model and Behaviour Change Wheel (BCW) can be used to understand behaviour in the context in which it occurs. The model states that Capability (C), Opportunity (O) and Motivation (M) each exert an influence on Behaviour (B) [36].

Dementia diagnosis comprises a set of behaviours; hence, to improve diagnosis and care, primary care practitioners need to change their behaviours [35]. In this study, the COM-B model is used to identify potential levers for behaviour change that support dementia diagnosis and to design a complex intervention for behaviour change for a geographical region. The application of the COM-B behaviour model involves three steps: (a) identification of barriers and facilitators to dementia diagnosis; (b) identification of appropriate behaviour change techniques to improve rates of dementia diagnosis; and (c) involvement of key stakeholders in the co-design of an intervention. Using the COM-B model, the barriers to dementia diagnosis are linked to specific behaviour change functions or strategies.

This study presents the design of the primary care practice change intervention arm of the Facing Dementia Together research project, which, as a whole, aims to increase dementia help-seeking and diagnosis [37]. The other arm involves a public campaign aimed at encouraging older adults and their families to seek timely help for dementia assessment [9]. Both interventions are being evaluated.

## 2. Methods

### 2.1. Aim

The aim of this study is to develop a complex intervention to improve rates of dementia diagnosis in Australian primary care, informed by behaviour change theory.

### 2.2. Design

The Facing Dementia Together practice change intervention development is based on primary research [18] and co-design workshops with key stakeholders, supplemented with a rapid literature review (unpublished). Co-design is the process of active collaboration between stakeholders in designing context-specific solutions to a prespecified problem [38]. The reporting of the study followed the Standards for Reporting Qualitative Research (SRQR) [39] (see Table S1).

#### 2.2.1. Using the BCW

**Figure 1:**
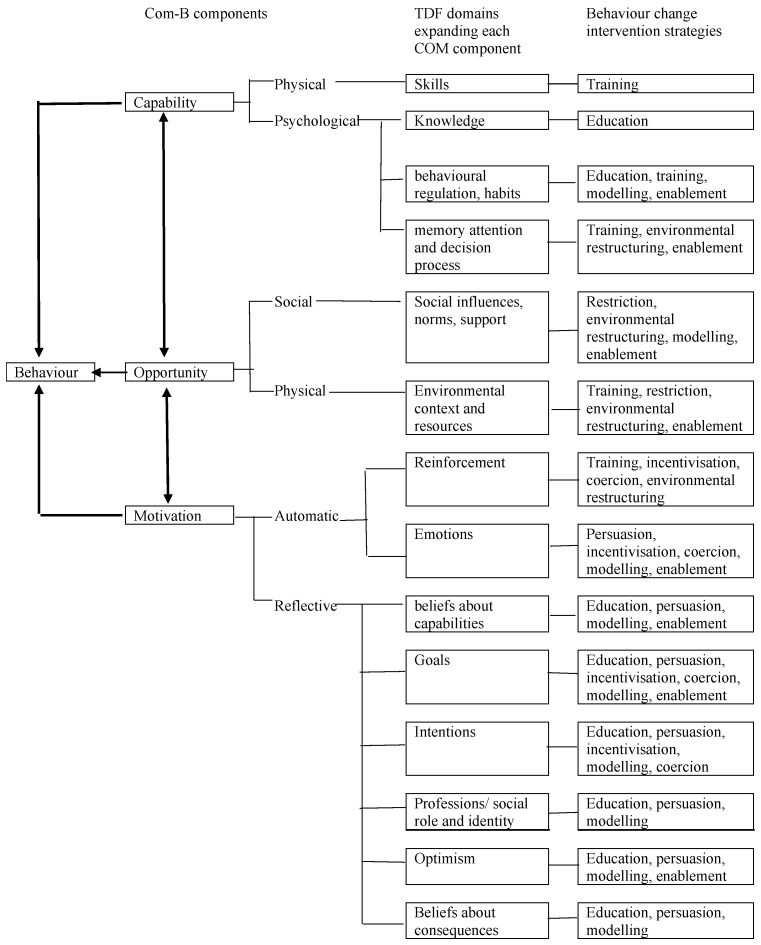
Links between Behaviour Change Wheel components—COM-B, TDF and behaviour change intervention strategies [36].

The BCW is made up of the COM-B, Theoretical Domains Framework (TDF) and nine intervention strategies [36]. The COM-B model is used as a starting point to understand behaviour in the context in which it occurs. The TDF builds upon the components of the COM-B model and outlines 14 types of barriers and facilitators that individuals may encounter. There are nine intervention strategies that drive behaviour change identified in the BCW. Each strategy targets different aspects of capability, opportunity, and motivation to effectively influence behaviour. Figure 1 illustrates the relationships between the COM-B, TDF and intervention strategies. Using the BCW allowed for a systematic approach in intervention development and evaluation.

#### 2.2.2. Setting

The study setting is general practices located in two Primary Health Network (PHN) regions in western Victoria and western Sydney. PHNs are regionally based, government-funded organisations that work to improve the efficiency, coordination and effectiveness of primary care. The PHNs were partners in the co-design and delivery of this intervention.

#### 2.2.3. Co-Design Participants

Purposeful and convenience sampling were used to identify and invite individuals with knowledge and experience in primary care in dementia and contextual knowledge of the intervention regions. The sample of primary care stakeholders, including GPs, general practice nurses (GPNs), practice managers and PHN practice facilitators, was recruited through the Western Victoria and Western Sydney PHNs, as well as the research team’s networks. GPNs work within general practices and assist GPs with patient care. Practice managers, who are often registered nurses, oversee the day-to-day operations of general practices. PHN practice facilitators support general practices in implementing quality improvement initiatives and accessing government incentives and funding, digital health tools, and professional development activities.

The research team sent each potential participant an email invitation to participate in the co-design process. This email invitation included a link to the online participant information and consent form (PICF) hosted on Qualtrics (Provo, UT, USA).

#### 2.2.4. Intervention Development Process

**Figure 2:**
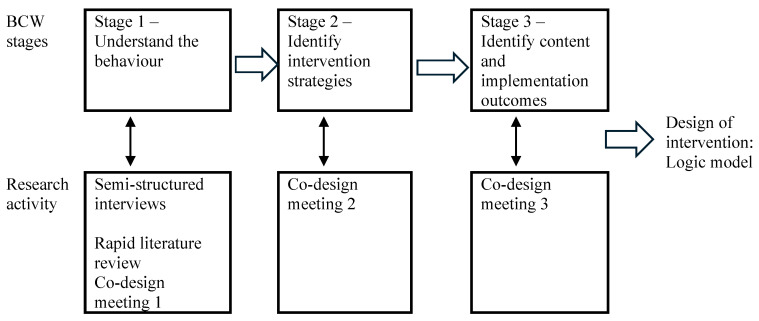
Facing Dementia Together practice change intervention development stages.

Development of the Facing Dementia Together practice change intervention was guided by the stages described in the BCW process [36]. The intervention development process is summarised in Figure 2.

The co-design process involved three one-hour meetings conducted online using the ZOOM platform (version 5.14). These meetings took place between May and July 2023.

#### 2.2.5. Foundational Research

Before co-design meeting 1, foundational research was carried out to understand the problem and identify what needs to change to increase rates of dementia diagnosis. The foundational research involved qualitative, semi-structured interviews with primary care stakeholders in both study locations. Its aim was to identify barriers to dementia diagnosis and provide a context for developing the Facing Dementia Together intervention [18]. A qualitative design with semi-structured interviews was conducted with 18 key primary care stakeholders recruited through the Western Victoria and Western Sydney PHNs. Four themes described current primary care practitioner practices in dementia diagnosis and management: (1) family concern triggers dementia investigation, (2) GPs delay conversations about dementia, (3) completing routine cognitive assessments in the 75+ health assessment, and (4) variability in post-diagnostic care.

A rapid review (unpublished) of the literature published between April 2013 and April 2023 on primary care best practices in dementia care was conducted. The review examined primary care interventions around dementia and identified intervention components and factors that influenced successful implementation and outcomes.

#### 2.2.6. Stage 1—Understand the Behaviour (Co-Design Meeting 1)

The first co-design meeting focused on the first stage of the BCW theoretical framework to ‘understand the behaviour’ [36]. During this meeting, participants were presented with the foundational research findings and the public help-seeking campaign [9] describing the barriers and facilitators to dementia diagnosis [18] to provide a context for the development of the intervention. The co-design participants then identified the target audiences for the intervention (i.e., GPs, GPNs, etc.) and the behaviours needed to increase dementia diagnosis rates. Behaviours were defined as functionally equivalent to specific actions [36]. The marketing and communication conceptual framework, “Know, Believe, Do” was used as a pragmatic approach to structure the discussion. The co-design group described what each audience needed to know to have the capability to support dementia diagnosis, what they needed to believe and or feel to motivate them to change behaviour, and what they needed to do and when they could do it. Current versus desired behaviours were explored. Following the co-design meeting, the research team summarised and categorised the discussion outcomes into the COM-B model.

#### 2.2.7. Stage 2—Identify Intervention Strategies (Co-Design Meeting Two)

The capabilities, opportunities, and motivations for each audience group were presented to the co-design group in meeting two for checking. Building on these, the co-design group provided input into the intervention’s main messages and generated ideas for activities to influence behaviour by each audience.

Following the second co-design meeting, the research team mapped the activities generated during the co-design meeting and identified from the literature review to the BCW intervention strategies [36]. This process identified the suite of intervention activities likely to bring about the desired behaviour change.

#### 2.2.8. Stage 3—Identify Content and Implementation Outcomes (Co-Design Meeting Three)

During the third co-design meeting, participants described in more detail and prioritised intervention activities. This included a discussion of promotional methods for the practice change intervention.

The research team worked with stakeholders to further detail the Facing Dementia Together practice change intervention activities, balancing costs, effectiveness and potential ongoing use of resources post-research project. The intervention activities were developed between January and September 2024.

Following the co-design meeting, the research team developed a logic model to describe the essential activities and mechanisms of the intervention. Logic models are recommended in the planning, implementation, and evaluation of complex interventions [40].

#### 2.2.9. Documentation and Prioritisation of Co-Design Decisions

Each co-design meeting was led by a facilitator (LF), and additional researchers (CG, MY, JJ) documented discussions and decisions. Priority areas for intervention development were sought through consensus within each meeting. The co-design meetings were audio-recorded to check the discussion content if required. A synthesis of the discussion in each co-design meeting was presented at the subsequent meeting and checked by participants for accuracy of interpretation and that decisions were captured accurately.

### 2.3. Reflexivity

The researchers are a multidisciplinary team with experience in primary care clinical dementia care, complex intervention design and/or dementia research: CG (PhD, primary care nurse, female); MY (MBBS, geriatrician, male); DP (PhD, general practitioner, female); SD (general practitioner, female); JJ (health researcher, female); LP (PhD, public health researcher, female); KL (PhD, occupational therapist, female); MG (PhD, occupational therapist, female); ET (PhD, pharmacist, male); HB (dementia researcher, psychiatrist, male); JS (PHN health analytics researcher, male); SF (health researcher, female); and LFL (PhD, psychologist, female). The team engaged in continuing discussions and challenged each other’s assumptions in developing the interventions.

### 2.4. Trustworthiness

Checking the interpretation of results was achieved by clarifying the meaning within the co-design discussions. A synthesis of the discussion in each co-design meeting was presented and checked for accuracy of interpretation with co-design participants in each subsequent meeting. An audit trail describing decision-making processes and outcomes was maintained throughout the intervention development process.

### 2.5. Patient and Public Participation

People living with dementia and their family carers contributed to the conceptualisation and design of the broader Facing Dementia Together project; however, they did not participate directly in this intervention design.

## 3. Results

### 3.1. Study Participants

A total of 15 individuals took part in co-design across three meetings, with individuals attending between one and three meetings, and each meeting having between eight and eleven participants. The study participants were primary care stakeholders (N = 15, 100%), including GPs (n = 5, 33%), GPNs (n = 4, 27%), a practice manager (n = 1, 6%), PHN practice facilitators (n = 3, 20%) and PHN Practice Facilitator Managers (n = 2, 13%). Participants came from Western Sydney (n = 7, 46%) and Western Victoria (n = 8, 53%). Ten (n = 10, 67%) participants identified as female, and five (n = 5, 33%) as male.

### 3.2. Stage 1—Understand the Behaviour

#### The Target Audience

GPs and GPNs were identified through co-design as the main audiences for the intervention. Changing the behaviour of these two groups was seen as having the greatest impact on increasing dementia diagnosis rates in primary care. Practice managers and receptionists were identified as secondary audiences who could support the practice change. Practice managers were described as the usual group who would oversee the implementation of practice improvement initiatives. Receptionists were identified as a secondary target audience as they have opportunities to notice patient behaviours that might indicate possible cognitive impairment.

PHN practice facilitators were identified as key stakeholders to drive change, as their role includes supporting practice improvement in primary care. This group was described as important in promoting the intervention, distributing resources and communicating intervention progress and outcomes.

A list of target behaviours that contribute to increasing the rate of dementia diagnosis for each audience group was generated. For the GP audience, these behaviours could be demonstrated by actions such as noticing potential cognitive changes, initiating conversations about cognitive changes, documenting the diagnosis of dementia in the patient’s medical record, referring to a specialist for assessment when needed, and communicating the diagnosis well. The capabilities, opportunities, and motivations to support each target audience to adopt the desired behaviours as part of the Facing Dementia Together practice change intervention are described in Table 1.

### 3.3. The Intervention Strategies

Six out of the nine intervention strategies described in the BCW (refer to Figure 1) are identified as relevant based on the outcomes of co-design and mapped from the COM-B. These intervention functions are education (increasing knowledge and understanding), training (imparting skills), persuasion (using communication to promote action), environmental restructuring (changing the physical or social context), enablement (increasing means and reducing barriers to the behaviour), and modelling (providing an observable example of the action) (Refer Table 2).

### 3.4. The Intervention Activities

The co-design participants generated a list of activities aimed at supporting the intervention audiences to engage in the behaviours that are predicted to contribute to increasing the rate of dementia diagnosis. Table 2 lists the intervention activities and how they align with the COM-B and selected BCW intervention strategies. The Facing Dementia Together practice change intervention is available on a website (https://facingdementiatogether.au/en/general-practice/) (accessed on 23 November 2025), which provides intervention information and access to the intervention’s resources.

Each activity included in the Facing Dementia Together practice change intervention covers multiple behaviour change strategies (refer to Table 2) and will be delivered concurrently. For example, peer-delivered online education sessions align with the behaviour change strategies of education and training, enablement, persuasion and modelling. Modelling and persuasion are addressed with the use of videos demonstrating health practitioner roles in the diagnosis of dementia. The main activity for each behaviour change strategy in the intervention is outlined below.

### 3.5. Online Dementia Education

An existing dementia in the primary care education program was identified. This education program is funded through Dementia Training Australia (DTA). DTA is a government-funded organisation that has been delivering dementia education for GPs and other members of the primary care team, notably GPNs, since 2013. This education program was identified as appropriate as it has a good reputation, is peer-led, is free of charge to attend, and contributes to GP and GPN Continuing Professional Development hours. The content and delivery of information were modified by the research team in collaboration with the DTA GP trainers based on the co-design outcomes. For example, content is included on why diagnosis is important even in late stages of dementia, the skills to have conversations about possible cognitive changes with a patient/family and managing difficult problems, such as driving and changed behaviour. Case studies and videos were included. Online delivery was selected in co-design as it was thought it would make it easier for time-poor primary care practitioners to attend.

The online dementia education primarily addressed ‘capability’ and aimed to increase audience knowledge and skills. Lack of knowledge and confidence in diagnosing dementia was recognised as a barrier to dementia diagnosis in the foundational research (Low et al., unpublished). In addition to ‘education’ and ‘training’, the online dementia education program aligned with several other behaviour change strategies as shown in Table 2. The use of peers to deliver education employs the behaviour change strategy of ‘persuasion,’ and the use of videos, such as a video of a GP ‘breaking the bad news’ to a patient, harnesses the behaviour change strategy of modelling. In the intervention, the education program is promoted and hosted by the PHN.

### 3.6. Geriatrician ‘Drop-In’ Support Sessions—Problem Solving with a Geriatrician

This activity aligned with the COM-B component ‘opportunity’ (social). It aims to provide GPs with an opportunity to discuss complex cases or raise diagnostic questions. The ZOOM platform will be used to optimise access, especially for GPs who are time-poor and geographically disparate. The barrier this intervention activity aims to mitigate is “uncertainty about diagnosis” by harnessing the intervention strategy “enablement” by providing support.

### 3.7. Practice Change Resources

A set of dementia diagnosis and management decision-making resources was developed, aligning with the COM-B components of capability (knowledge) and opportunity (physical). The corresponding intervention strategies are education and enablement. The resources include a ‘brain health checklist’, ‘pre-diagnosis management checklist’, post-diagnosis management checklist’, decision chart for when to refer to a specialist’, ‘take-home post-diagnostic patient information’, and ‘receptionist information on managing patients with cognitive changes’.

Practice development resources, including quality improvement templates and a business case describing the opportunity to improve practices around dementia, will also be included. A ‘Medicare Benefits Schedule (MBS) item pathway for dementia diagnosis and care’ was provided to demonstrate opportunities for funded dementia care to address the barrier of a lack of funded time to provide dementia care. The MBS is a listing of the healthcare services subsidised by the Australian Government.

The resources will be available on the Facing Dementia Together practice change website. Printed versions of the resources will also be available. The version tailored to general practices in the Western Victoria PHN can be viewed at https://facingdementiatogether.au/en/wp-content/uploads/sites/2/2024/05/Face-Dementia-GP-Resource-Pack-Western-Victoria.pdf (accessed on 23 November 2025).

### 3.8. Short Informative Articles in PHN Newsletter

The PHNs email an online newsletter to all general practices fortnightly. The newsletter includes healthcare information, available opportunities and new resources. In co-design, three topics relevant to GP practice were identified as commonly challenging. These topics are (1) Mild Cognitive Impairment in General Practice, (2) BPSD in General Practice, and (3) Driving and Dementia in General Practice. The goal is to offer practical advice on these topics, supporting capability (knowledge) using the behaviour change function of education. Each article includes a QR code to the Facing Dementia Together website. The articles are brief, at 300 words in length, and written in a conversational tone.

### 3.9. Stand-Alone Short Videos

Four stand-alone YouTube videos were made for the Facing Dementia Together practice change intervention website. They are titled, (1) ‘Detecting dementia—The role of medical receptionists’ (2:58), (2) ‘Supporting people living with dementia—The role of medical receptionists’ (2:30), (3) ‘Detecting dementia—Raising the issue in general practice’ (5:25) and (4) ‘Driving and dementia in general practice’ (5:30). These videos featured a GP, GPN, practice manager, receptionist and geriatrician, all from Western Victoria.

The purpose of the first three videos is to provide education to support a whole-of-practice approach in dementia diagnosis, describing how GPs, GPNs and receptionists all have a role. The COM-B component is motivation (professional role and identity). In co-design, receptionists were recognised as having an essential role in supporting patients who present with cognitive changes. Two videos are directed to the receptionist to increase capability (knowledge) in recognising cognitive changes, strategies to support people with cognitive changes, and communicating concerns to the GP and or GPN. The fourth video provided education to increase GPs’ capability (knowledge and skills) to have a conversation on driving and dementia. This topic was identified in the foundational research as a barrier to diagnosis, as GPs were reluctant to raise this topic [18].

### 3.10. Tailored Data—Auditing and Benchmarking

**Figure 3:**
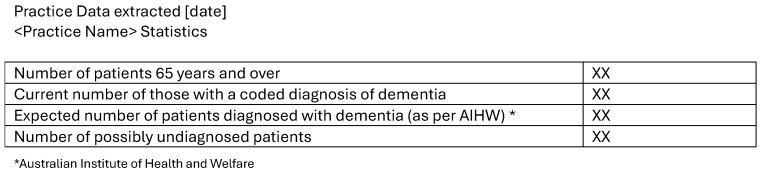
Auditing and benchmarking report.

General practices will be able to review their own documented record of dementia diagnoses and compare this to the expected prevalence using data from the Australian Institute of Health and Welfare (AIHW) [41] by provision of a PHN-generated report. Figure 3 shows the report that the general practice will receive. This will be accompanied by an invitation to the education program and links to Facing Dementia Together resources.

This intervention activity supports dementia diagnosis through the COM-B component ‘motivation’ (belief about capabilities). It uses the intervention strategy of ‘persuasion’ by using a credible source and feedback to show that the GP can make a difference to the diagnosis of dementia. The auditing and benchmarking activity also aligns with ‘social opportunity’ (norms) by providing a comparison with prevalence data and motivation (reinforcement) through supporting new habits.

### 3.11. Risk Alert in Electronic Medical Record

This invention activity was available for Western Victoria PHN general practices only. A pop-up Population Level Analysis and Reporting (POLAR) data tool was developed by the Western Victoria PHN, providing a dementia risk alert for a patient using the Walrus point-of-care tool. Walrus is a point-of-care tool that prompts the GP about missing data, clinical prompts and risk scores for the patient on-screen. If the patient meets the criteria to be considered for a dementia risk assessment, the dementia risk tool alerts the GP via a pop-up notification. Depending on the patient’s age and indigenous status, a combination of factors will trigger the Dementia Prompt, namely, active smoker, alcohol consumption of 10+ drinks per week, diabetes Type 1 (active or inactive) or Type 2 diagnosis (active), and an active diagnosis of depression or atrial fibrillation or stroke or hypertension or insomnia or traumatic brain injury. The combination of data to inform the triggering of the risk alert tool is adapted from the validated CogDrisk risk assessment tool. The CogDrisk assessment tool assesses individual exposure to risk factors known to be associated with an increased risk of developing dementia [42]. GPs are given the option to follow a link to the full CogDrisk assessment tool for use with or by patients when a risk for dementia has been identified by the dementia risk tool.

This intervention activity aligns with the COM-B component capability (memory, attention and decision-making processes) and the intervention strategy ‘environmental restructuring’ as it provides an on-screen prompt. The risk alert tool will support GPs being alert to patients with chronic conditions being at a higher risk of developing dementia.

This intervention activity will not be developed in the Western Sydney PHN setting, as general practices in this region use different software incompatible with the risk alert being developed for use in Western Victoria. It was cost-prohibitive within this research project to develop the risk alert in the other software platform.

### 3.12. Logic Model

**Figure 4:**
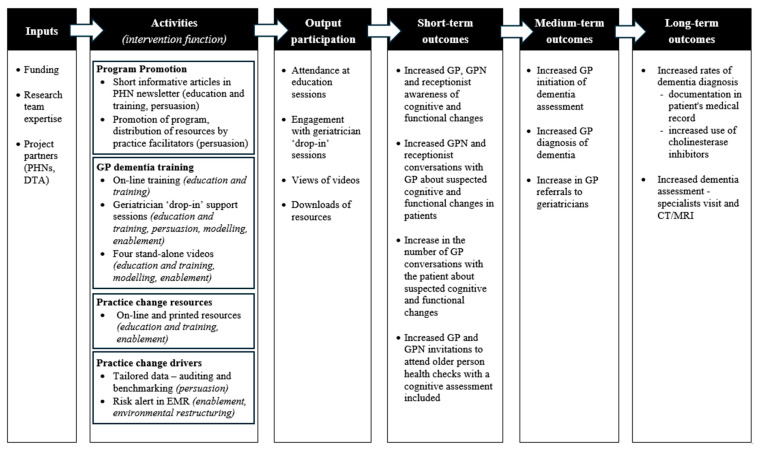
Logic model for Facing Dementia Together Practice Change Intervention Program.

The essential elements and mechanisms of the Facing Dementia Together practice change intervention were brought together and are presented in the logic model shown in Figure 4. The logic model describes the outputs and projected outcomes of the intervention and will be used to guide implementation and evaluation.

## 4. Discussion

This study used a co-design process to develop a theoretically informed complex intervention aimed at increasing the rate of dementia diagnosis in primary care. Six behaviour change strategies were identified—education, training, enablement, modelling, persuasion, and environmental restructuring—using the COM-B model and BCW, to include in the intervention. Co-design prioritised seven activities to include in the Facing Dementia Together practice change intervention—peer-led on-line dementia education and training, geriatrician ‘drop-in’ on-line support sessions, quality improvement in dementia care sessions, stand-alone videos, auditing and benchmarking, a dementia risk alert tool, and a set of dementia diagnosis and management decision-making resources. It is reassuring to note that these behaviour change activities are consistent with effective strategies identified in the literature. What sets the Facing Dementia Together practice change intervention apart is its use of multiple strategies simultaneously, rather than relying on just one.

Education was included as it is an essential element for facilitating behavioural change, although it is often not sufficient on its own [43]. Education and training activities in the intervention comprised elements known to be effective. For instance, peer-led online sessions utilising practice-relevant case studies have been shown to increase participants’ self-reported knowledge and confidence regarding dementia care [44]. The online GP-led education and training sessions are supplemented with written resources and videos. Interactive educational outreach has been widely used to promote evidence-based clinical practice, and peer-to-peer education has proven effective in fostering stakeholder engagement and commitment to practice changes [45].

The videos outlined the opportunities of various primary care team members, including GPs, GPNs and receptionists, to support dementia diagnosis. These videos serve a dual purpose: providing information and modelling the desired behaviours to be adopted within primary care teams. This approach is supported by social learning theory, which posits that people acquire new behaviours, attitudes, and emotional reactions by observing others within a social context [46]. Modelling was employed in the intervention to reinforce the practical application of improved dementia diagnosis practices within a collaborative team approach.

All members of the primary care team were included in the education program, as interprofessional education contributes to enhanced teamwork and collaboration [47]. Team-based care is a foundational element of high-performing primary care [48] and was described as a facilitator to dementia diagnosis in the foundational research [18].

Clinical pathways were included in the intervention resources to support the provision of integrated care. The inclusion of this component is supported by evidence that practice change is enhanced if team members have a clear knowledge of who does what, when, where and how [12].

Persuasion and enablement were identified as important behaviour change functions to be included in the Facing Dementia Together practice change intervention. Provision of tailored data, auditing and benchmarking has been shown to prompt health practitioners to modify their practice when provided data showing that their clinical practice is inconsistent with a desirable target [49]. Using patient documentation tools, such as prompts and risk alerts, has been shown to enhance practitioner awareness, support clinical decision-making, and improve communication across the care team [12].

An intervention’s impact relies on how well it is implemented; thus, implementation outcomes are key to achieving the desired clinical or service changes [50]. Following the introduction of the Facing Dementia Together practice change intervention, the implementation outcomes, for example, site-specific and digital implementation variability, will be systematically assessed. Such evaluations provide insight into how and why implementation strategies either succeed or fail, particularly within complex health contexts [36].

### Limitations and Strengths

The study was set in two Australian regions, potentially limiting the generalisability of findings. However, one setting was regional, and the other was a metropolitan multi-cultural setting, which is a strength. Additionally, a strength of the study was the development of an intervention tailored to local barriers to dementia diagnosis in primary care.

Using a relatively small purposive and convenience sample may limit generalisability to all practices, but every participant had prior experience in primary care, enabling them to reflect and offer valuable insights into the research question. The study did not gather information about participants’ levels of practitioner experience or their previous training in dementia, which could have contributed further to the findings. Despite this, all participants shared experiences from their own backgrounds, which supported strong internal validity, and there was a high level of agreement among the participants.

This paper details the multiple small decisions that were made during iterative co-design to develop a practice change intervention. Describing these processes transparently helps other researchers developing primary care interventions understand the rationale for the intervention components and may help them decide whether they are relevant for their context [51]. Co-design, purposively with primary care practitioners and members of organisations who promote practice improvement, helps ensure that the intervention will be both acceptable and sustainable. Consumers were not directly involved in the design of the primary care practice change intervention; however, the barriers they experienced [9] informed this study’s co-design process.

## 5. Conclusions

Dementia frequently goes undiagnosed, and national and international dementia action plans have highlighted the importance of supporting GPs in diagnosing and managing dementia. Our theory-informed, co-designed program was developed to address the multiple barriers to dementia identification and assessment in primary care in a manner that is potentially implementable across a region. The next step is to implement and evaluate.

## Figures and Tables

**Table 1:** COM-B for each audience for the Facing Dementia Together practice change program.

Audience	Capability What Knowledge/Skills Are Needed to Do Target Behaviours?	Opportunity What Are the Opportunities to Adopt the Target Behaviours:	Motivation What Beliefs/Feelings/Intentions/Goals Lead to Adoption of the Target Behaviours:	Behaviour What Action Demonstrates the Behaviour
General practitioners	Have the skills to have conversations about possible cognitive changes with a patient/family Understand why conversations about brain health are important Understand why diagnosis, even in moderate to late stages of dementia is important Know how to undertake assessments for dementia and when to refer to specialist Have the skills to communicate diagnosis well Know how to treat and manage MCI or dementia, as well as manage other chronic conditions when the person has dementia Have knowledge of the service system to support management and support of people with cognitive changes and family	All patient contacts, including when the presenting issue does not relate to cognition or function Government rebated team-based chronic condition management care plans Government rebated routine older person health assessments (include cognitive assessments as usual practice) Health policy and community expectations that diagnosis and management of dementia can occur in primary care	I have an important role as a GP to assess, diagnose and help manage dementia Treating and managing dementia makes a difference to my patients’ lives I have the skills to raise cognitive and functional changes with my patients I have the support I need to diagnose and manage dementia I have a range of support services to refer my patients and their families to The PHN will support my training and resource needs for assessment and management of dementia	Notice potential cognitive changes Initiate conversations about cognitive changes Document diagnosis of dementia in patient medical record Refer to a specialist for assessment when needed Communicate diagnosis well Make appropriate referrals for treatments, supports and care for patients with MCI or dementia and carers Work collaboratively with the practice team to support dementia assessment and management
General practice nurses	Know the signs that may indicate dementia Have the knowledge and skills to assess for cognitive changes Understand the value of including the family in gathering information about cognitive change Know the available support services and referral pathways	All patient contacts, including when the presenting issue does not relate to cognition or function Government rebated team-based chronic condition management care plans Government rebated routine older person health assessments (include cognitive assessments as usual practice) Health policy and community expectations that diagnosis and management of dementia can occur in primary care	I have an important role in noticing cognitive changes in my patients Managing dementia makes a difference to my patients’ lives I have the skills to raise cognitive and functional changes with my patients I have a range of support services to refer my patients and their families to The PHN will support my training and resource needs for assessment and management of dementia	Notice potential cognitive changes Informally assess for cognitive/functional changesassessments in all patient contacts Initiate conversation about suspected cognitive changes with GP and patients Use older person health assessments to complete cognitive assessment tolls Develop and follow up on dementia informed chronic disease management plans
Practice Managers	Understand the important role that the General Practice team plays in detecting, diagnosing and managing dementia Know that the Face Dementia practice change program is available in region	PHN promotions and interactions	Dementia is a priority for primary care There is a benefit for the practice team members and patients, to participate in the practice change program	Sign practice up for Facing Dementia Together practice change program Support the General Practice team to reflect, monitor and improve practice around dementia.
Receptionists	Know the signs that may indicate cognitive changes Have the skills to communicate appropriately with the patient with cognitive changes.	Interactions with patients and families when making appointments and payments, and managing patient requests (e.g., for scripts)	I have a role in primary care team in noticing cognitive changes I am confident I can communicate with clinicians if I observe cognitive or functional difficulties in a patient	Be alert for potential cognitive changes Talk with GP/nurse about cognitive changes Use strategies to communicate effectively with patients with cognitive difficulties

**Table 2:** Using the BCW to link the COM-B component and behaviour change strategies with intervention activities.

COM-B Component	Theoretical Domain (TDF) Describing COM Component	Target Audience	Examples (From Table 1)	BCW—Intervention Strategy	Facing Dementia Together Practice Change Intervention Activity
Capacity	Physical capacity				
	Physical skills	GP	Dementia assessment skills	Training	On-line dementia education—embedded videos and case studies
		GPN	Dementia assessment skills	Training	On-line dementia education—embedded videos and case studies
	Psychological capacity				
	Knowledge	GP	Knowing how to diagnose and manage dementia	Education	On-line dementia education Short informative articles in PHN newsletter
		GPN	Recognising signs of cognitive change	Education	On-line dementia education
		Receptionist	Know the signs that may indicate cognitive change	Education	Stand-alone short videos
	Cognitive & interpersonal skills	GP	Initiating potentially difficult conversations	Training	On-line dementia education—videos and case studies
		GPN	Asking questions about cognitive change	Training	DTA on-line dementia education—videos and case studies
		Receptionist	Skills to communicate concerns with GP/GPN	Training	Stand-alone short video of a receptionist discussing communication strategies
	Memory, attention & decision-making processes	GP	Some patients have chronic conditions putting them a higher risk of developing dementia	Environmental restructuring	Risk alert in EMR
	Behavioural regulation, habits	GP	Knowing what to do in dementia diagnosis and management	Modelling	On-line dementia education—embedded video demonstrating desired behaviour by a GP
Opportunity	Physical opportunity				
	Environmental context and resources	GP, GPNs, Practice Manager	Government rebated routine older person health assessments include cognitive assessments as usual practice	Enablement	Resources—quality improvement templates, business case PHN practice change intervention promotion as an opportunity to improve practice around dementia
		GP	Appointment when presenting issue does not relate to cognition or function Have a trigger/prompt	Environmental restructuring	Risk alert in EMR
	Social opportunity				
		GP, GPN	Dementia diagnosis in primary care to be usual practice	Environmental restructuring	Tailored data—Auditing and benchmarking
		GP	Have support from others—not alone	Enablement	Geriatrician drop-in support sessions
Motivation	Reflective motivation				
	Professional role and identity	GP, GPN, Receptionist	I have a role in assessment, diagnosis and management of dementia	Education	On-line dementia education sessions Stand-alone short videos
	Beliefs about capabilities	GP, GPN	Confidence that I can make a difference	Persuasion	Tailored data—Auditing and benchmarking
	Optimism	GP, GPN	Confidence that person living with dementia/family can access appropriate services	Education	On-line dementia education sessions
	Beliefs about consequences	GP, GPN	Belief that making a diagnosis is beneficial	Education	On-line dementia education sessions
	Automatic motivation				
	Reinforcement	GP, GPNs, Practice Manager	Gap in care—diagnoses rates in the GP Clinic are less than expected	Environmental restructuring	Tailored data—Auditing and benchmarking

## Data Availability

The raw data supporting the conclusions of this article will be made available by the authors upon request.

## Supplementary Materials

The following supporting information can be downloaded at: https://www.mdpi.com/article/10.3390/ijerph23050653/s1, Table S1: Standards for Reporting Qualitative Research (SRQR) [39].

## Abbreviations

The following abbreviations are used in this manuscript:

AIHW	Australian Institute of Health and Welfare
BPSD	Behaviours and psychological symptoms of dementia
BCW	Behaviour Change Wheel
COM-B	Capability, Opportunity, Motivation-Behaviour
GP	General practitioner
GPN	General Practice Nurse
DTA	Dementia Training Australia
PHN	Primary Health Network
SRQR	Standards for Reporting Qualitative Research

## References

[1] Mazza D., McCarthy E., Camões-Costa V., Mansfield E., Bryant J., Waller A., Lin X., Piterman L. (2021). Prioritising national dementia guidelines for general practice: A Delphi approach. Australas. J. Ageing.

[2] Frost R., Rait G., Wheatley A., Wilcock J., Robinson L., Harrison Dening K., Allan L., Banerjee S., Manthorpe J., Walters K. (2020). What works in managing complex conditions in older people in primary and community care? A state-of-the-art review. Health Soc. Care Community.

[3] Prince M., Comas-Herrera A., Knapp M., Guerchet M., Karagiannidou M. (2016). Improving Healthcare for People Living with Dementia.

[4] Breithaupt A.G., Sideman A.B., Goode C., Hill-Sakurai L., Premkumar M., Scheffler A.W., Chodos A., Tsoy E., Rankin K.P., Kramer J.H. (2025). Enhancing early detection of cognitive impairment in primary care with the TabCAT-BHA. Alzheimer’s Dement..

[5] Lang L., Clifford A., Wei L., Zhang D., Leung D., Augustine G., Danat I.M., Zhou W., Copeland J.R., Anstey K.J. (2017). Prevalence and determinants of undetected dementia in the community: A systematic literature review and a meta-analysis. BMJ Open.

[6] Dobson A.J., Flicker L., Almeida O.P., Waller M., Anstey K. (2023). Different estimates of the prevalence of dementia in Australia, 2021. Med. J. Aust..

[7] Mansfield E., Noble N., Sanson-Fisher R., Mazza D., Bryant J. (2018). Primary Care Physicians’ Perceived Barriers to Optimal Dementia Care: A Systematic Review. Gerontologist.

[8] Garrett M.H., Azar D., Goeman D., Thomas M., Craig E.A., Maybery D. (2024). Health and social care needs of people living with dementia: A qualitative study of dementia support in the Victorian region of Gippsland, Australia. Rural Remote Health.

[9] Low L.-F., Barcenilla-Wong A., Laver K., Yates M., Gibson C., Shen S., Hall D., Brodaty H., Pond D., Comans T. (2025). Development of a model of help-seeking for dementia diagnosis by the person experiencing changes and family supporters. Aging Ment. Health.

[10] Cox C.G., Brush B.L., Kobayashi L.C., Roberts J.S. (2025). Determinants of dementia diagnosis in U.S. primary care in the past decade: A scoping review. J. Prev. Alzheimer’s Dis..

[11] Aminzadeh F., Molnar F.J., Dalziel W.B., Ayotte D. (2012). A Review of Barriers and Enablers to Diagnosis and Management of Persons with Dementia in Primary Care. Can. Geriatr. J..

[12] Arsenault-Lapierre G., Le Berre M., Rojas-Rozo L., McAiney C., Ingram J., Lee L., Vedel I. (2022). Improving dementia care: Insights from audit and feedback in interdisciplinary primary care sites. BMC Health Serv. Res..

[13] Fox C., Maidment I., Moniz-Cook E., White J., Thyrian J.R., Young J., Katona C., Chew-Graham C.A. (2013). Optimising primary care for people with dementia. Ment. Health Fam. Med..

[14] Bernstein-Sideman A., Ma M., Hernandez de Jesus A., Alagappan C., Razon N.a., Dohan D., Chodos A., Al-Rousan T., Alving L.I., Segal-Gidan F. (2023). Primary Care Pracitioner Perspectives on the Role of Primary Care in Dementia Diagnosis and Care. JAMA Netw. Open.

[15] Visser F.C.W., van Eersel M.E.A., van der Zaag-Loonen H.J., Hempenius L., Perry M., van Munster B.C. (2024). Doing the Right Thing? General Practitioners’ Considerations in Achieving a Timely Dementia Diagnosis. Int. J. Geriatr. Psychiatry.

[16] Low L.-F., McGrath M., Swaffer K., Brodaty H. (2019). Communicating a diagnosis of dementia: A systematic mixed studies review of attitudes and practices of health practitioners. Dementia.

[17] Balsinha C., Iliffe S., Dias S., Freitas A., Barreiros F.F., Gonçalves-Pereira M. (2022). Dementia and primary care teams: Obstacles to the implementation of Portugal’s Dementia Strategy. Prim. Health Care Res. Dev..

[18] Low L.-F., Gibson C., Barcenilla-Wong A., Daniel S., Phillipson L., Gresham M., Laver K., Tan E., Daly S., Pond C.D. (2026). How does Australian primary care face dementia? Identifying current practice and barriers in dementia diagnosis and management in Australian primary care—A qualitative interview study. BMC Prim. Care.

[19] Irish Department of Health (2014). The Irish National Dementia Strategy.

[20] Department of Health (2009). Living Well with Dementia: A National Dementia Strategy.

[21] Australian Department of Health, Department of Health and Ageing (2024). National Dementia Action Plan 2024–2034.

[22] Public Health Agency of Canada (2019). A Dementia Strategy for Canada.

[23] Cartz-Piver L., Calvet B., Mehrabian-Spassova S., Raycheva M., Rejdak K., Papuk E., Leperre-Desplanques A., Krolak-Salmon P. (2023). Empowering general practitioners in dementia care: The ANTISTIGMA education intervention in Europe. Int. J. Geriatr. Psychiatry.

[24] Perry M., Drašković I., Lucassen P., Vernooij-Dassen M., van Achterberg T., Rikkert M.O. (2011). Effects of educational interventions on primary dementia care: A systematic review. Int. J. Geriatr. Psychiatry.

[25] Pond D., Mate K., Stocks N., Gunn J., Disler P., Magin P., Marley J., Paterson N., Horton G., Goode S. (2018). Effectiveness of a peer-mediated educational intervention in improving general practitioner diagnostic assessment and management of dementia: A cluster randomised controlled trial. BMJ Open.

[26] Flodgren G., Eccles M.P., Shepperd S., Scott A., Parmelli E., Beyer F.R. (2011). An overview of reviews evaluating the effectiveness of financial incentives in changing healthcare professional behaviours and patient outcomes. Cochrane Database Syst. Rev..

[27] Thyrian J.R., Hertel J., Wucherer D., Eichler T., Michalowsky B., Dreier-Wolfgramm A., Zwingmann I., Kilimann I., Teipel S., Hoffmann W. (2017). Effectiveness and Safety of Dementia Care Management in Primary Care: A Randomized Clinical Trial. JAMA Psychiatry.

[28] Callahan C.M., Boustani M.A., Unverzagt F.W., Austrom M.G., Damush T.M., Perkins A.J., Fultz B.A., Hui S.L., Counsell S.R., Hendrie H.C. (2006). Effectiveness of collaborative care for older adults with Alzheimer disease in primary care: A randomized controlled trial. J. Am. Med. Assoc..

[29] Gandolfi S., Bellè N., Nuti S. (2025). Please mind the gap between guidelines & behavior change: A systematic review and a consideration on effectiveness in healthcare. Health Policy.

[30] Iliffe S., Robinson L., Bamford C., Waugh A., Fox C., Livingston G., Manthorpe J., Brown P., Stephens B., Brittain K. (2014). Introducing case management for people with dementia in primary care: A mixed-methods study. Br. J. Gen. Pract..

[31] Disler R., Pascoe A., Anderson H., Piejko E., Asaid A., Disler P. (2022). A new model for general practice-led, regional, community-based, memory clinics. BMC Prim. Care.

[32] Dodd E., Cheston R., Fear T., Brown E., Fox C., Morley C., Jefferies R., Gray R. (2014). An evaluation of primary care led dementia diagnostic services in Bristol. BMC Health Serv. Res..

[33] Skivington K., Matthews L., Simpson S.A., Craig P., Baird J., Blazeby J.M., Boyd K.A., Craig N., French D.P., McIntosh E. (2021). A new framework for developing and evaluating complex interventions: Update of Medical Research Council guidance. BMJ.

[34] Moore G.F., Audrey S., Barker M., Bond L., Bonell C., Hardeman W., Moore L., O’Cathain A., Tinati T., Wight D. (2015). Process evaluation of complex interventions: Medical Research Council guidance. BMJ Br. Med. J..

[35] Foy R., Francis J.J., Johnston M., Eccles M., Lecouturier J., Bamford C., Grimshaw J. (2007). The development of a theory-based intervention to promote appropriate disclosure of a diagnosis of dementia. BMC Health Serv. Res..

[36] Michie S., Atkins L., West R. (2014). The Behaviour Change Wheel: A Guide to Designing Interventions.

[37] Facing Dementia Together. Facing Dementia Together. https://facingdementiatogether.au/en/.

[38] Vargas C., Whelan J., Brimblecombe J., Allender S. (2022). Co-creation, co-design, co-production for public health—A perspective on definitions and distinctions. Public Health Res. Pract..

[39] O’Brien B.C., Harris I.B., Beckman T.J., Reed D.A., Cook D.A. (2014). Standards for reporting qualitative research: A synthesis of recommendations. Acad. Med..

[40] Skivington K., Matthews L., Simpson S.A., Craig P., Baird J., Blazeby J.M., Boyd K.A., Craig N., French D.P., McIntosh E. (2021). Framework for the development and evaluation of complex interventions: Gap analysis, workshop and consultation-informed update. Health Technol. Assess..

[41] Australian Institute of Health and Welfare (2025). Dementia in Australia.

[42] Anstey K.J., Kootar S., Huque M.H., Eramudugolla R., Peters R. (2022). Development of the CogDrisk tool to assess risk factors for dementia. Alzheimers Dement..

[43] Arlinghaus K., Johnston C. (2017). Advocating for Behavior Change With Education. Am. J. Lifestyle Med..

[44] Foley T., Jennings A., Boyle S., Smithson W.H. (2018). The development and evaluation of peer-facilitated dementia workshops in general practice. Educ. Prim. Care.

[45] Luetsch K., Wong G., Rowett D. (2023). A realist synthesis of educational outreach visiting and integrated academic detailing to influence prescribing in ambulatory care: Why relationships and dialogue matter. BMJ Qual. Saf..

[46] Horsburgh J., Ippolito K. (2018). A skill to be worked at: Using social learning theory to explore the process of learning from role models in clinical settings. BMC Med. Educ..

[47] Jennings A., McLoughlin K., Boyle S., Thackeray K., Quinn A., O’Sullivan T., Foley T. (2019). Development and evaluation of a primary care interprofessional education intervention to support people with dementia. J. Interprof. Care.

[48] Ghorob A., Bodenheimer T. (2015). Building Teams in Primary Care: A Practical Guide. Fam. Syst. Health.

[49] Ivers N., Yogasingam S., Lacroix M., Brown K.A., Antony J., Soobiah C., Simeoni M., Willis T.A., Crawshaw J., Antonopoulou V. (2025). Audit and feedback: Effects on professional practice. Cochrane Database Syst. Rev..

[50] Proctor E., Silmere H., Raghavan R., Hovmand P., Aarons G., Bunger A., Griffey R., Hensley M. (2011). Outcomes for implementation research: Conceptual distinctions, measurement challenges, and research agenda. Adm. Policy Ment. Health.

[51] Hoddinott P. (2015). A new era for intervention development studies. Pilot Feasibility Stud..

